# Influence of indirect air-cooling on the variation in intrapulpal temperature during rapid high-intensity light-curing using a bulk-fill resin composite

**DOI:** 10.4317/jced.62228

**Published:** 2025-01-01

**Authors:** Samille Biasi Miranda, Vinícius Cristovão de Oliveira Mendes, Caroline de Farias Charamba Leal, Luiz Antonio Soares Falson, Guilherme da Luz Silva, Ana Karina Maciel de Andrade, Rodrigo Barros Esteves Lins, Marcos Antônio Japiassú Resende Montes

**Affiliations:** 1 Departament of Restorative Dentistry, School of Dentistry, University of Pernambuco, Recife 50100-130, PE, Brazil; 2 Department of Semiology and Clinical, School of Dentistry, Federal University of Pelotas, Pelotas, RS, Brazil; 3 Department of Oral Medicine, School of Dentistry, Postgraduate Program in Dentistry, Federal University of Rio Grande do Sul, Porto Alegre, RS, Brazil; 4 Department of Restorative Dentistry, School of Dentistry, Federal University of Paraíba, João Pessoa 58051-900, PB, Brazil; 5 Department of Restorative Dentistry, School of Dentistry, Federal University of Alagoas, Maceió 57072-900, AL, Brazil; 6 Department of Dental Materials, School of Dentistry, University of Pernambuco, Recife, PE, Brazil

## Abstract

**Background:**

This study investigated the effect of indirect air-cooling on the variation in intrapulpal temperature (ΔT) during different light-curing protocols of bulk-fill resin composites in molars with class V cavities.

**Material and Methods:**

A nonretentive class V cavity was prepared in a maxillary molar. The intrapulpal temperature changes with and without indirect air-cooling were recorded using a type K thermocouple. The cavity was restored with bulk-fill resin composites: Tetric PowerFlow (Ivoclar, Vivadent) and Filtek Bulk-Fill Flow (3 M, ESPE). The tooth was exposed to different light-curing protocols (n=5) using the Valo Grand light-curing unit: (i) high-intensity light-curing protocol of 3200 mW/cm² (3 and 6 s) and (ii) moderate-intensity light-curing protocol of 1000 mW/cm² (10 and 20 s). The ΔT data were subjected to two-way ANOVA, followed by the Bonferroni post hoc test.

**Results:**

A significant increase in temperature was observed with the use of the high-intensity light-curing resin composite without indirect air-cooling. In addition, the application of air-cooling significantly decreased the temperature in all the groups except for the Tetric PowerFlow bulk-fill resin composite at 3 s of light-curing.

**Conclusions:**

Indirect air-cooling resulted in a lower increase in intrapulpal temperature during the light-curing of bulk-fill resin composites, making it an effective alternative technique for controlling the temperature rise in class V restorations with 1 mm of remaining dentin.

** Key words:**Dental curing lights, dental pulp, temperature, resin composite.

## Introduction

Resin composites are widely used because of their esthetics, tissue preservation, and mechanical capabilities, which play a significant role in patients self-esteem (1,2). The constant effort of professionals to reduce clinical steps has driven the development of bulk-fill resin composites, which optimize the restorative protocol by being placed in the dental cavity in a single increment of up to 5 mm thickness, providing greater depth of polymerization (3) and reducing the sensitivity of the incremental technique (4). This improvement has been made possible by the presence of stress-reducing monomers and new photoinitiators with high initiation efficiency, which together promote better light absorption and stress distribution from contraction (5).

The light-curing of resin composites plays a crucial role in the clinical success of restorations, directly influencing their final properties (2). The use of light-emitting diodes (LEDs) as a light source in light-curing units (LCUs) has become the most common method for polymerizing restorative materials (6). Recently, high-power LED-LCUs have been introduced to increase the intensity of emitted light, allowing for their application in an ultra-rapid curing protocol (7,8). This protocol involves the use of high radiant emittance levels (up to 3000–3500 mW/cm²) combined with a reduced exposure time (1-3 s) (9,10).

This approach offers advantages such as reduced clinical time and technique simplification, preventing operational errors (11). However, initially, very short light exposure was not sufficient to allow proper polymerization of resin composites, prompting manufacturers to develop a restorative material compatible with this protocol (10). Thus, the composition of bulk-fill resin composites can be modified to allow their use with ultra-rapid curing through the incorporation of reversible addition–fragmentation chain transfer (RAFT) molecules, which control the polymerization reaction (11), and the refinement of their photoinitiator system (12).

The new bulk-fill resin composites consist of more cross-linked polymer networks with short and homogeneous chains, which undergo gelation at higher degrees of conversion, resulting in lower contraction forces (11,12). Despite these resins exhibiting good properties in combination with ultra-fast light-curing, the high levels of radiant emittance have raised concerns about biocompatibility, as they can lead to temperature increases, posing risks to soft and pulpal tissues (13). This is particularly due to the light emitted by the LED-LCU, the volume of the restorative material, and the temperature generated during the exothermic reaction of light-curing, which can cause changes in the intrapulpal temperature (14).

An increase of 5.5°C has been reported as the maximum that pulpal tissue can tolerate, as exceeding this limit can lead to irreversible pulpitis (15). Previous studies have revealed an increase in the intrapulpal temperature in class V cavities and dentin discs restored with new bulk-fill resin composites and the ultra-rapid curing protocol (14,16). One alternative to reduce the thermal stress generated in the pulp during light-curing is the use of air-cooling via a three-way syringe. This cooling may be beneficial in clinical situations involving high radiant emission (17). However, studies related to methods for reducing or controlling this heat increase in the pulp are still limited. To date, no study has investigated the impact of indirect air-cooling during the light-curing of bulk-fill resin composites specifically designed for high-intensity protocol.

The objective of this study was to evaluate the effectiveness of indirect air-cooling on intrapulpal temperature changes during different curing protocols of bulk-fill resin composites in molars with class V cavities. This could contribute to safer and more effective clinical guidelines for this new type of bulk-fill resin composite. The null hypothesis was that the increase in intrapulpal temperature is greater without air-cooling than with air-cooling during exposure to the LED-LCU.

## Material and Methods

-Ethical considerations

This study was approved by the Research Ethics Committee of the University of Pernambuco (CEP) in accordance with Resolution No. 466/12 of the National Health Council under CAAE number 66493323.6.0000.5207. A healthy extracted maxillary molar from the University’s Tooth Bank was used for all experiments. The inclusion criterion was that the molar be intact and indicated for extraction for therapeutic reasons (e.g., orthodontic needs or nonerupted teeth). Teeth with caries, cracks, fractures, or existing restorations were excluded from the sample.

-Study design

The sample consisted of 40 samples that were randomly assigned by a single operator into eight groups (n=5) on the basis of curing time (3, 6, 10, and 20 s), light intensity (3200 and 1000 mW/cm²), type of bulk-fill resin composite (Tetric PowerFlow and Filtek Bulk‒Fill Flow), and the presence or absence of air-cooling during curing. The sample size was determined on the basis of a previous study that also evaluated temperature changes during high-intensity curing protocols (14).

-Cavity Preparation

A nonretentive class V cavity with divergent walls (2 mm width, 2 mm depth, and 5 mm length) was prepared in a molar tooth using a cylindrical diamond bur number 1014 (KG Sorensen, Cotia, SP, Brazil) at high speed with constant water/air-cooling. The cavity dimensions were verified using a calibrated periodontal probe and a specimen. The remaining dentin in the prepared cavity was approximately 1 mm. The cavity walls were polished with finishing and polishing tips (American Burrs) to avoid mechanical retention on the cavity walls and to facilitate the removal of the restorative material. The tooth roots were sectioned 4 mm below the cementoenamel junction and enlarged with Gates Glidden drills (Dentsply Sirona, York, PA, USA).

-Restorative Procedure

Bulk-fill resin composites Tetric PowerFlow (Ivoclar Vivadent AG, Schaan, Liechtenstein) and Filtek Bulk-Fill Flow (3 M ESPE, St. Paul, USA) were used ( Table 1). To facilitate the reuse of the dental cavity, the tooth element was isolated with a thin layer of vaseline inside the cavity. The resin composites were then placed into the class V cavity in a single increment, following the manufacturer’s instructions, using a resin applicator tip. The materials were subsequently light-cured with a broad-spectrum LED-LCU (Valo Grand, Ultradent, South Jordan, USA), as outlined in  Table 2.

A high-intensity light-curing protocol (3200 mW/cm²) with short exposure times (3 and 6 s) and moderate-intensity mode (1000 mW/cm²) with longer exposure times (10 and 20 s) were used. Details of the materials used in the study are presented in  Table 1. The irradiance of the LED-LCU was confirmed before conducting the experimental tests using a spectrometer (RedTide USB650, OceanOptics, Florida, USA) calibrated with NIST laboratory references (Managing Accurate Resin Curing (MARC) System; Bluelight Analytics Inc., Halifax, Canada) before and after each test session.

In all the evaluations, the LED-LCU tip was positioned in contact (0 mm) with the vestibular surface of the tooth. For experimental tests where bulk-fill resin composites were light-cured with indirect air-cooling, a three-way syringe from the University of Pernambuco’s Dental Clinic was used at a distance of 2 cm from the class V restoration site during the curing protocol. The bulk-fill resin composites were removed between the temperature measurements, and a new portion of uncured restorative material was placed.

Intrapulpal temperature evaluation

Temperature evaluation was conducted following an adapted methodology from previous studies (14,16,18). The temperature changes were recorded using a digital thermometer connected to a type K thermocouple with a datalogger (Instrutherm, São Paulo, Brazil) with a resolution of 0.1°C. For temperature analysis, a type K thermocouple wire was inserted into the pulp chamber of the tooth with expanded roots close to the class V cavity.

**Figure 1:**
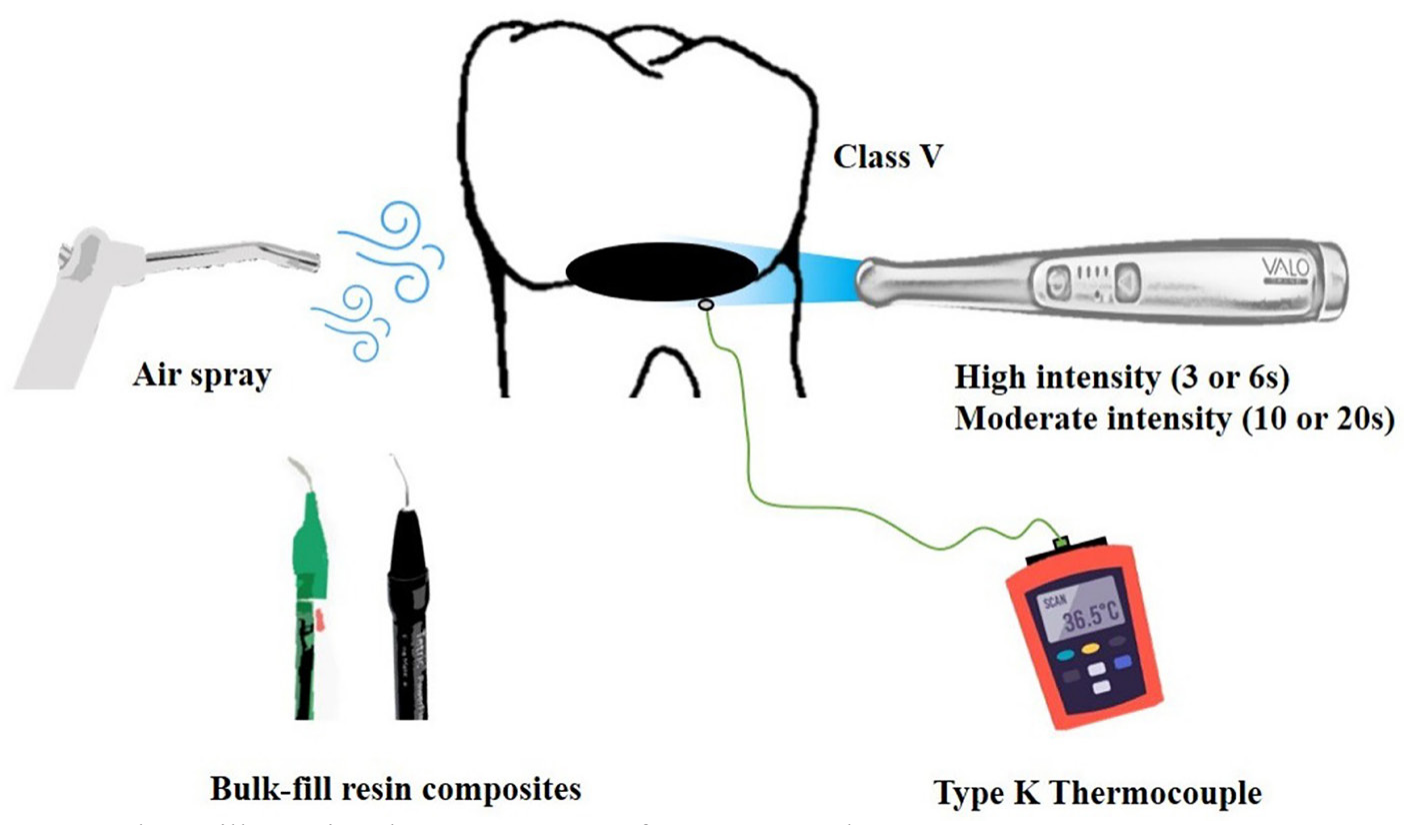
Scheme illustrating the measurement of temperature changes.

The temperatures of all the samples were measured in a controlled environment with a constant temperature of 27°C by a single trained and calibrated operator. The type K thermocouple used to measure the temperature was positioned inside the pulp chamber, juxtaposed to the wall facing the class V cavity and verified radiographically. The mean temperature change was calculated as the difference between the ambient temperature and the temperature recorded after the experiments. The scheme illustrating how the temperature was recorded is shown in Figure 1.

-Statistical analysis

The data obtained were tabulated in a custom spreadsheet (Excel, Microsoft, Redmond, WA, USA). Data normality was assessed using the Shapiro‒Wilk and Levene tests, followed by two-way ANOVA. Post hoc analysis was conducted using the Bonferroni test, with a predefined significance level of 0.05. Statistical calculations for analyzing differences between groups were performed using SPSS software (Statistical Package for the Social Sciences, USA).

## Results

 Table 3 shows the temperature difference (ΔT) recorded during the light-curing protocols in the four different modes at various time intervals (3, 6, 10, and 20 s), with and without simultaneous indirect air-cooling. When the class V cavity was exposed to light with the high-intensity curing protocol (3 and 6 s), the temperature increased with increasing exposure time (TPF-3 s, 6.70°C; TPF-6 s, 12.86°C). However, when an air spray was applied for cavity cooling during light-curing, the heat increase was less pronounced than that under no cooling (SprayTPF-3 s, 6.48°C; SprayTPF-6 s, 6.80°C).

In the groups where the class V cavity was exposed to light with the moderate-intensity curing protocol (10 and 20 s), the temperature demonstrated the same trend of increasing heat with increasing exposure time (FBF-10 s, 8.80°C; FBF-20 s, 10.30°C). Additionally, with air-cooling, there was a substantial reduction in the intrapulpal heat increase (SprayFBF-10 s, 4.98°C; SprayFBF-20 s, 5.88°C).

The greatest temperature change was recorded in the TPF-6 s group, followed by the FBF-20 s group, indicating a proportional relationship between the temperature increase and the exposure time for both the resin composites and the curing protocols. Statistical differences were found between the groups for TPF-3 s and TPF-6 s (*p*<0.001), TPF-6 s and SprayTPF-6 s (*p*<0.001), and FBF-20 s and SprayFBF-20 s (*p*<0.001).

## Discussion

Dental restorations can impair pulp health due to tissue dehydration, instrument vibration, and temperature increases caused by light curing, which are mainly caused by the heat emitted by the light curing unit (LCU) (19). The null hypothesis that the intrapulpal temperature increase is greater without air-cooling than with air-cooling during LED-LCU exposure was accepted. The data found in this study are consistent with a previous study in which a three-way syringe effectively reduced the intrapulpal temperature during light-curing of restorative material (18). Therefore, the use of air during light-curing effectively removes hot air from the vicinity of the restored dental element, making it more effective at reducing the intrapulpal temperature than light is at increasing it (20).

Despite the reduction in temperature with the use of air-cooling, most temperatures still exceed 5.5°C, the limit established for maintaining pulpal vitality (15). A similar result was reported by Miranda *et al*. (2024), who also investigated intrapulpal temperature during ultra-fast light-curing of modified bulk-fill resins and reported that an increase in intrapulpal temperature could be harmful to the pulp. This phenomenon may be related to the fact that bulk-fill resins tend to be more translucent, which can facilitate the conduction of heat toward the pulp (20). Additionally, this study was conducted in class V cavities, which typically have a thin layer of remaining dentin, resulting in less thermal insulation. It has been demonstrated that as dentin thickness decreases from 1.0 mm to 0.5 mm, there is a significant increase in intrapulpal temperature, highlighting the relationship between the amount of remaining dentin and heat transmission to the intrapulpal region (21).

In our study, we used a molar tooth, and it was possible to observe an association between the increase in intrapulpal temperature and dental volume, suggesting that teeth with smaller volumes tend to experience a greater increase in temperature (18). However, in our research, by using the same type of cavitated tooth to measure intrapulpal temperature increase during restorative procedures, we eliminated any bias related to differences in volume among the sample teeth. Intrapulpal temperature is also influenced by microcirculation; lower temperatures are observed when microcirculation in the pulpal tissues is simulated, likely because the blood aids in dissipating the heat generated by the curing unit (22). In our study, this simulation was not performed, reflecting a more clinically challenging situation, similar to what is encountered when restorative procedures are performed with a local anesthetic containing a vasoconstrictor (18).

Controlling heat during restorative procedures to ensure pulp integrity has been a significant concern (23). Indirect air-cooling is an easily applicable technique that does not compromise the properties of the resin and is an important tool in clinical practice, especially in challenging situations, such as class V cavities with minimal remaining dental tissue (18,20). An *in vivo* study evaluating intact premolars revealed that when air spray was applied before starting the light-curing protocol and maintained during light exposure, the temperatures of the enamel and dentin were reduced, corroborating our findings (20).

The reduction in temperature on the walls of the pulpal chamber establishes a gradient that facilitates the thermal conduction of heat from the pulp toward the walls. This characteristic is crucial, as it indicates the persistence of a residual effect even after the air flow is interrupted, since heat distribution continues until dynamic thermal equilibrium is achieved (20). Therefore, air-cooling during light-curing is recognized in the literature as the most effective method to mitigate heat generation, although excessive dehydration of the dental element must be avoided (24).

This study has several limitations, as it is an *in vitro* study with standardized and controlled conditions during the experiment. Temperature dissipation in a vital tooth may differ from that observed in an extracted tooth, especially due to the presence of pulpal microvascularization, which was not simulated in this study. Additionally, the ideal 0 mm distance between the restoration and the light-curing unit recommended in this study may not always be clinically feasible. Temperature measurement was performed at only one point; it would be interesting to conduct measurements at multiple points and consider the application of dentin adhesive. We recommend conducting randomized clinical trials for a more comprehensive understanding of the topic. However, this study provides valuable knowledge and encourages the use of indirect air-cooling during light-curing, a simple practice that can be incorporated into dental practice routines. This approach helps reduce the risks associated with pulp exposure to high-power LED equipment, making the clinical use of modified bulk-fill resins safer.

## Conclusions

The use of indirect air-cooling with a three-way syringe resulted in a lower increase in the intrapulpal temperature during the light-curing of the bulk-fill resin composites, regardless of the intensity and exposure time of the LED-LCU. In teeth with class V cavities that have minimal dentin remaining, air-cooling can be an effective alternative for controlling the rise in intrapulpal temperature.

## Figures and Tables

**Table 1:** Characteristics of the materials used according to the manufacturer.

Material	Type	Manufacturer	Detalhes
Filtek Bulk-Fill Flow Resin	Bulk-Fill Flow resin, color A1	3 M ESPE, St.Paul, EUA	Bis-Ema, Bis-Gma, UDMA, Treated silanized ceramic, Benzotriazole, Substituted dimethacrylate, TEGDMA, Ytterbium fluoride.
Tetric PowerFlow Resin	Bulk-Fill Flow resin, IVA color	Ivoclar Vivadent AG, Schaan, Liechtenstein	Dimethacrylates, Barium Glass, Ytterbium Trifluoride and Copolymers.
Valo Grand Cordless	LED Polywave	Ultradent, South Jordan, EUA	Tip Diameter (12 mm) Wavelength (385 – 515 nm)

**Table 2:** Experimental groups for temperature analysis.

Group	Time (s)	Irradiance (mW/cm^2^)	Protocol
TPF-3 s	3	3200	Light-curing of Tetric PowerFlow bulk-fill resin composite
TPF-6 s	6	3200	Light-curing of Tetric PowerFlow bulk-fill resin composite
FBF-10 s	10	1000	Light-curing of Filtek Bulk-Fill Flow bulk-fill resin composite
FBF-20 s	20	1000	Light-curing of Filtek Bulk-Fill Flow bulk-fill resin composite
SprayTPF-3 s	3	3200	Air-cooling + Light-curing of Tetric PowerFlow bulk-fill resin composite
SprayTPF-6 s	6	3200	Air-cooling + Light-curing of Tetric PowerFlow bulk-fill resin composite
SprayFBF-10 s	10	1000	Air-cooling + Light-curing of Filtek Bulk-Fill Flow bulk-fill resin composite
SprayFBF-20 s	20	1000	Air-cooling + Light-curing of Filtek Bulk-Fill Flow bulk-fill resin composite

**Table 3:** Mean and standard deviation (SD) of the intrapulpal temperature difference (ΔT - °C) under different light-curing protocols, with and without indirect air-cooling.

Group	Spray	Temperature (ΔT)		
		3 s/3,200 mW/cm^2^	6 s/3,200 mW/cm^2^	p-valor
Tetric PowerFlow	No	6.70 (1.4) aB	12.86 (0.8) aA	< 0.001
		6.48 (0.4) aA	6.80 (0.4) bA	0.568
p value	Yes	0.694	< 0.001	
		10 s/1,000mW/cm^2^	20 s/1,000mW/cm^2^	
Filtek Buk-Fill Flow	No	8.80 (0.6) aA	10.30 (1.8) aA	0.062
		4.98 (0.8) bA	5.88 (1.1) bA	0.246
p value	Yes	< 0.001	< 0.001	

Two-way ANOVA with Bonferroni post hoc. Lowercase letters indicate statistical differences between rows (comparing with or without spray at the same time/irradiance). Uppercase letters indicate statistical differences between columns (comparing time/irradiance with or without spray).

## Data Availability

The datasets used and/or analyzed during the current study are available from the corresponding author.
